# A Macro-to-Micro Framework for Bridge-Deck Pavement-Distress Inspection Using LiDAR-Based Screening and YOLOv8s Image Detection: A Case Study of the Jingzhou Yangtze River Highway Bridge and Public Benchmark Data

**DOI:** 10.3390/s26196217

**Published:** 2026-09-30

**Authors:** Yadong Huang, Ying Chang, Di Deng, Li Lu, Shuting He, Jianghua Liu, Shengjun Deng

**Affiliations:** 1 College of Water Conservancy and Hydropower Engineering, Hohai University, Nanjing 210098, China; 2 Hubei Provincial Communications Planning and Design Institute Co., Ltd., Wuhan 430051, China; 3 College of Transportation Engineering, Nanjing Tech University, Nanjing 211816, China; 4 School of Water Transport and Land Transportation Engineering, Wuhan Technical College of Communications, Wuhan 430065, China; 5 China Electronics Standardization Institute Huadong Branch, Suzhou 215100, China

**Keywords:** airborne LiDAR, UAV imagery, bridge deck pavement, suspected-region screening, pavement-distress detection, YOLOv8s

## Abstract

Timely bridge-deck pavement inspection must cover large areas while preserving the image detail needed to recognize local distress. This study develops a macro-to-micro workflow with separate LiDAR screening and YOLOv8s image-detection branches. Airborne LiDAR data from the Jingzhou Yangtze River Highway Bridge supported deck extraction and geometric screening. Processing included statistical outlier removal, coordinate normalization, voxel sampling, local plane fitting, residual and normal-discrepancy screening, and spatial clustering. Deck extraction retained 179,914 of 311,784 representative points. Geometric screening based on residual and normal discrepancy identified 17,075 suspected points. Region filtering followed by bounding-box export yielded 423 points in seven candidate regions for targeted inspection. The 2025 inspection report documented pavement distress, and site personnel confirmed corresponding distress within the screened regions. The image branch used the official image-level splits of UAV-PDD2023. The epoch-146 checkpoint achieved the highest validation mAP50 and was evaluated on the official-test split. Official-test precision reached 0.85591, with a recall of 0.85882, mAP50 of 0.88556, and mAP50–95 of 0.58195. The model detected all six pavement-distress classes under the fixed benchmark protocol. The two branches support wide-area geometric screening and detailed image analysis for bridge-deck pavement inspection.

## 1. Introduction

Access equipment and traffic control complicate repeated bridge-deck surveys by increasing labor demands and traffic exposure. Inspector-dependent condition ratings add another source of variability to repeated surveys. Spencer et al. (2019) and Luo et al. (2023) reviewed non-contact alternatives based on computer vision and 3D sensing [1,2]. Unmanned aerial vehicles (UAVs) extend visual inspection to hard-to-access bridge areas and retain surface texture for visible-damage assessment [3,4]. Direct spatial coordinates from UAV imagery require external positioning or three-dimensional reconstruction.

Deep learning has been applied to component degradation prediction, as illustrated by the WPD and DPNN approach reported by Hong et al. [5]. Image-based defect detection has shifted from thresholding and edge-based extraction toward convolutional neural networks (CNNs), which learn task-specific crack features from training data [6]. One-stage and region-based detectors have been tested on concrete bridge surfaces [7,8], and road-surface studies have used bounding-box annotations to detect multiple distress classes [9,10]. Reviews identify object-scale variation, background complexity, dataset coverage, and transfer between inspection scenarios as continuing limitations [11,12,13]. Defect appearance also changes with viewpoint, camera-to-surface distance, illumination, platform motion, and sensor noise [14]. YOLOv8 variants have been tested on road damage and bridge cracks under complex backgrounds [15,16,17,18]. Feature-enhancement and dual-network designs target difficult cracks and bridge-surface defects [19,20]. Huyan et al. developed CrackDN for sealed and unsealed crack detection under complex backgrounds [21]. Outside pavement inspection, Liu et al. investigated multifeature attention for object detection in aerial imagery [22].

Image-based analysis also supports applications beyond defect detection. Sovanneth et al. combined YOLOv8 with the Segment Anything Model for pavement crack quantification [23]. Optical character recognition linked the results to geographic coordinates. Obunguta et al. connected image-processing outputs with a probabilistic pavement management model [24]. Zheng et al. used multistage semisupervised active learning to reduce annotation demands and connect bridge crack identification with segmentation and width measurement [25].

For large bridges, planning where to acquire detailed images is an additional inspection task. Wide-area UAV surveys also record intact pavement and look-alike patterns, including shadows, lane markings, and expansion-joint edges. Reviewing all of these images increases the manual workload and creates more opportunities for false detections. LiDAR geometry can prioritize candidate regions before detailed UAV imaging. LiDAR point clouds record bridge-deck elevation, surface continuity, and local geometric variation. These properties have supported bridge condition assessment [26] and curvature-based anomaly screening [27]. Zhou et al. (2022) described laser and visual sensing as complementary sources of geometry and texture [28]. The fusion of UAV LiDAR and images has also been investigated for locating bridge damage [29].

Raw engineering LAS files rarely contain only the bridge-deck surface. Noise and returns from adjacent components can distort local neighborhoods, while large coordinate magnitudes complicate numerical processing. Preprocessing reduces these effects but does not make texture-dominated cracks geometrically observable; narrow cracks may still produce elevation changes too weak for confirmation from point-cloud geometry alone. The proposed workflow uses bridge-scale geometry to prioritize areas for detailed visual inspection. The Jingzhou case quantifies this spatial screening output, while a separate six-class image benchmark characterizes the detector used in the proposed workflow.

**Figure 1:**
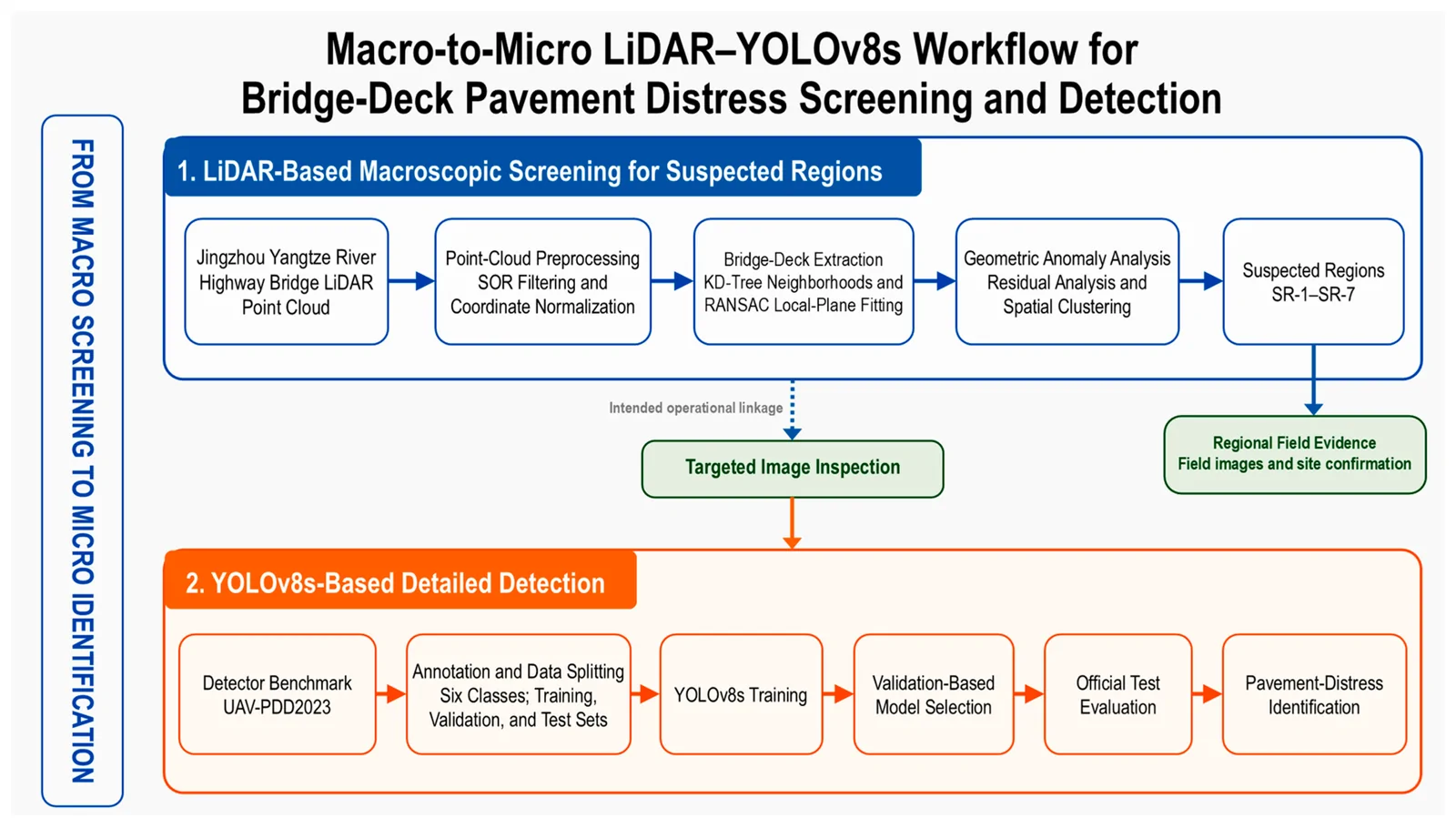
Proposed macro-to-micro inspection framework. LiDAR (Livox Technology, Shenzhen, China) screens the entire bridge deck to identify candidate regions for targeted image inspection. The dashed connector denotes the intended operational linkage. In this study, the LiDAR and YOLOv8s stages were evaluated separately using the Jingzhou point cloud and UAV-PDD2023, respectively.

The framework uses bridge-scale geometry to identify candidate regions for targeted image inspection (Figure 1). The Jingzhou LiDAR case and the UAV-PDD2023 benchmark evaluate the screening and image-detection stages, respectively. In the engineering branch, LiDAR data from the Jingzhou Yangtze River Highway Bridge were used to extract the deck surface and screen candidate regions showing local geometric anomalies. In the benchmark branch, YOLOv8s was trained on UAV-PDD2023, selected using its published validation split, and evaluated once on the official-test split. Source-group overlap among the published image-level splits was audited separately.

## 2. Materials and Methods

### 2.1. Engineering Case and Data Organization

The Jingzhou Yangtze River Highway Bridge provided the engineering case, and Table 1 assigns separate analytical roles to three data sources. The engineering LiDAR dataset contained 10,374,647 raw LAS points. These points supported deck extraction, plane-residual analysis, and candidate-region screening.

The 2025 periodic inspection report recorded alligator cracking, potholes, and a transverse crack using defect identifiers and photographs. Site personnel responsible for the inspection confirmed that corresponding pavement distress was present within the LiDAR-derived candidate regions. Together, these sources support the interpretation that the candidate regions contained pavement distress. The available evidence lacks coordinate-tagged documentation for auditable one-to-one matching between individual defects and SR-1–SR-7. UAV-PDD2023 contained 2440 annotated image tiles and 11,158 annotated instances across six pavement-distress classes. The dataset supported detector training, validation-based checkpoint selection, and one evaluation on the official-test split. It was not used to assess the LiDAR screening step.

### 2.2. LiDAR-Based Macroscopic Screening

LiDAR-based macroscopic screening was used to extract the bridge-deck area and provide a spatial reference for targeted image inspection. Let the original point cloud be defined as

*P* = {*p_i_*∣ *p_i_* = (*x_i_*, *y_i_*, *z_i_*), *i* = 1,2, …,*N*}, (1)

where *p_i_* denotes the *i*-th point, (*x_i_*, *y_i_*, *z_i_*) denotes its spatial coordinates, and *N* is the total number of points. Because airborne or mobile LiDAR data may contain scattered noise caused by platform motion, environmental interference, reflection differences, and ranging errors, the point cloud was first filtered before local geometric analysis.

#### 2.2.1. Statistical Outlier Removal (SOR)

SOR is a point-cloud filtering method that identifies and removes outliers based on the statistical distribution of local neighborhood distances. This algorithm was used to remove isolated noise points. Its basic assumption is that normal bridge-deck or bridge-body points maintain relatively stable distances to neighboring points, whereas outlier points have significantly larger neighborhood distances. For a point *p_i_*, let *N_k_*(*p_i_*) denote its set of *k* nearest neighbors, and let *p_ij_* denote the *j*-th nearest neighbor of *p_i_*. The mean neighborhood distance is

(2) $${\overset{–}{d}}_{i}=\frac{1}{k}\sum_{j=1}^{k}{\left\|p_{i}-p_{\mathrm{ij}}\right\|}_{2}.$$

After the global mean *μ* and standard deviation *σ* of all ${\overset{–}{d}}_{i}$ are calculated, the outlier threshold is defined as

*T*_SOR_ = *μ* + *ασ*. (3)

**Figure 2:**
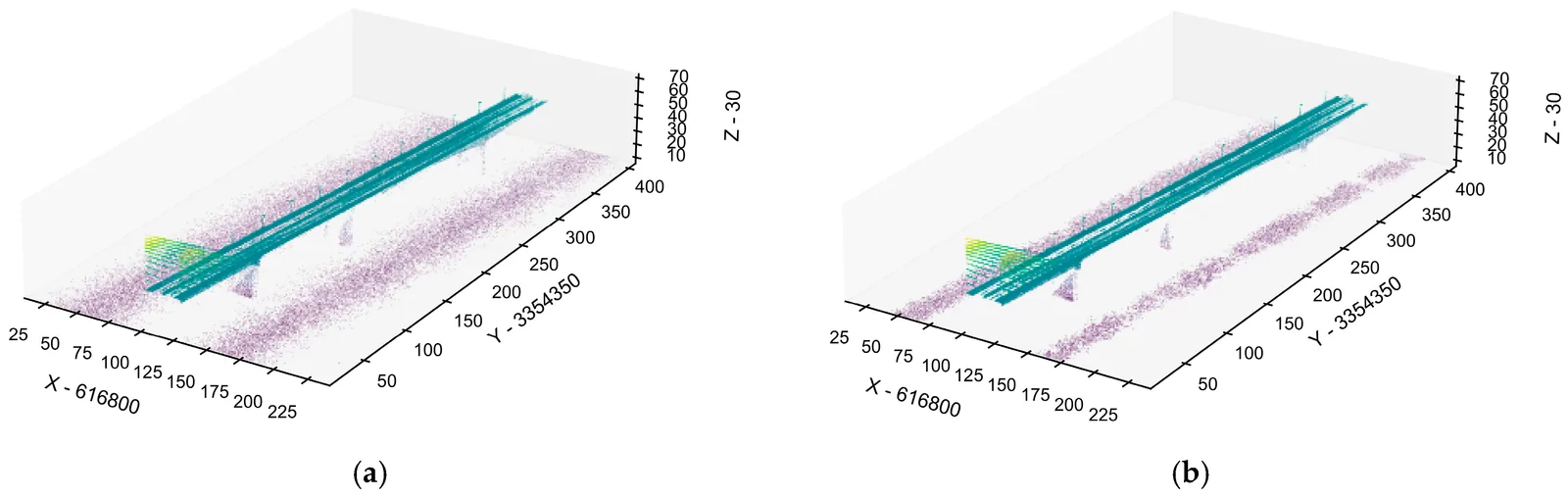
Comparison of the point clouds before and after SOR filtering: (**a**) original point cloud; (**b**) point cloud after SOR filtering. Coordinates are expressed in the original LAS coordinate units. Green points correspond to the bridge structure, while purple points represent the surrounding non-bridge/background point cloud.

If $\overset{–}{d_{i}}$ > *T*_SOR_, the point is regarded as an outlier and removed; otherwise, it is retained. In this study, *k* = 50 and *α* = 0.8 defined the SOR configuration used for the Jingzhou point cloud. A local one-factor-at-a-time sensitivity analysis showed that, with *α* = 0.8, *k* = 40, 50, and 60 yielded 2, 7, and 6 candidate regions, respectively; with *k* = 50, *α* = 0.7, 0.8, and 0.9 yielded 3, 7, and 5 candidate regions, respectively. Under the adopted setting, SOR removed 14.05% of the input points while retaining 8,916,957 points for subsequent processing. With the other processing parameters held fixed, the five SOR settings yielded 166,748–184,013 effective-deck points, 15,880–17,386 suspected points, and 2–7 candidate regions. The local analysis quantifies how SOR settings affect the candidate-region output and provides a reference for parameter assessment in this case. SOR is a common point-cloud preprocessing method, and related implementations are widely used in the Point Cloud Library [31]. Figure 2 compares the point cloud before and after SOR filtering.

#### 2.2.2. Coordinate Normalization

After filtering, the point cloud may still be expressed in a large engineering or geographic coordinate system. Directly using such coordinates for neighborhood search, local plane fitting, and residual calculation may increase numerical instability. Therefore, the geometric center of the filtered point cloud was calculated as

(4) $$C=\left(\overset{–}{x},\overset{–}{y},\overset{–}{z}\right)=\frac{1}{N_{\mathrm{SOR}}}\sum_{p_{i}\in P_{\mathrm{SOR}}}p_{i},$$

where *P*_sor_ denotes the filtered point cloud. Each point was then translated to a local coordinate system:

(5) $$p_{i}^{\prime }=p_{i}-C.$$

In this study, scale normalization was applied after centroid translation. The scale factor was defined as the maximum Euclidean distance of any translated point from the origin:

(6) $$D_{\max}={\underset{i}{\max}\left\|p_{i}^{\prime }\right\|}_{2},{\tilde{p}}_{i}=\frac{p_{i}^{\prime }}{D_{\max}}.$$

For the filtered point cloud, the calculated scale factor was *D_max_* = 225.7839 in the original LAS coordinate units. The normalization produced dimensionless coordinates while preserving the relative geometry of the point cloud. All subsequent voxel sizes and distance-based thresholds were therefore applied in the normalized coordinate system. The centroid *C* and scale factor *D_max_* were retained to map the results back to the original engineering coordinate system.

#### 2.2.3. Local Neighborhood Construction and Plane Fitting

The normalized point cloud was voxel-subsampled using cubic voxels with a side length of 0.0015 normalized units, retaining one representative point per occupied voxel. This retained 311,784 representative points from 8,916,957 filtered points (3.50%), reducing the number of local plane fits required for geometric screening. A K-D tree was constructed from the retained points, and the 50 nearest neighbors of each point defined its local neighborhood. Within the bridge-deck pavement area, each local surface was approximated by a plane as follows:

*Ax* + *By* + *Cz* + *D* = 0. (7)

The distance residual from point *p_i_* = (*x_i_*, *y_i_*, *z_i_*) to the fitted plane is

(8) $$r_{i}=\frac{\left|Ax_{i}+By_{i}+Cz_{i}+D\right|}{\sqrt{A^{2}+B^{2}+C^{2}}}.$$

This residual reflects local geometric deviation from an approximately flat deck surface. To reduce the influence of residual noise, bridge-edge points, and local anomalies, random sample consensus (RANSAC) was used for local plane estimation. The RANSAC distance threshold was set to 0.003 in normalized coordinate units, and the maximum number of iterations was set to 15. The 0.003 tolerance defines the inlier band for local plane fitting and equals twice the voxel side length.

#### 2.2.4. Bridge-Deck Extraction and Suspected-Region Screening

The effective-deck area was extracted from the normalized representative point cloud using the distance to a robustly estimated reference deck plane. Points with a plane distance not exceeding 0.025 in normalized coordinate units were retained as effective-deck points. For these points, the residual and normal-vector discrepancy thresholds were defined as the 95th percentiles of their respective distributions. Geometrically suspected points were then selected as

*R*_sus_ = {*p_i_* ∈ *P*_deck_|*r_i_* ≥ *T_r_* ∨ Δ*θ_i_* ≥ *T_θ_*}, (9)

where *R*_SUS_ is the set of geometrically suspected points, *P_deck_* is the effective-deck point cloud, *r_i_* is the local plane residual, and Δ*θ_i_* is the local normal-vector discrepancy. The resulting thresholds were *T_r_* = 0.0009166206 in normalized coordinate units and *T_θ_* = 1.3159202 rad. Spatial clustering linked suspected points within a radius of 0.006 in the normalized horizontal plane and retained connected components containing at least 30 points. Components with a maximum horizontal extent above 0.15 normalized units or a horizontal aspect ratio above 12 were excluded. The maximum number of retained regions was set to eight; seven regions remained after clustering and filtering in this case. For each retained region, all suspected points within its saved XY bounding box were exported. These regions represent local geometric anomalies for targeted inspection rather than confirmed distress.

The 95th-percentile rule selects the upper tail of each geometric-feature distribution, with numerical cutoffs calculated from the extracted deck points. These geometric settings were held fixed in the local SOR sensitivity analysis.

To quantify spatial coverage, the evaluated domain D was defined as the union of occupied cells in the normalized XY plane. The grid had a cell width of 0.0015 and origin (0, 0); occupied cells contained at least one effective-deck point. Empty cells were not filled. Candidate regions were represented by their saved axis-aligned XY bounding boxes, without display padding. The candidate-area fraction was the area of D intersected with the union of these bounds, divided by the area of D. The area-reduction fraction was one minus this value. D represents point-cloud-supported coverage rather than a surveyed deck boundary.

### 2.3. YOLOv8s-Based Pavement-Distress Detection

The inspection-report photographs were excluded from detector evaluation because their viewpoints, resolution, compression, scale, and camera calibration were not standardized. Detector performance was evaluated separately on the public UAV-PDD2023 benchmark. The dataset contains six annotated pavement-distress classes and published image-level splits, enabling evaluation under a fixed training and testing protocol.

#### 2.3.1. Detection Task and Dataset

The public benchmark was UAV-PDD2023 [30], which contains 2440 annotated image tiles and 11,158 annotated distress instances. Its six classes are alligator crack, longitudinal crack, oblique crack, pothole, repair, and transverse crack. These class labels were retained without merging them into a generic candidate category.

The published image-level lists were preserved, giving 1561 training images, 391 validation images, and 488 official-test images. Their corresponding annotation counts were 7084, 1723, and 2351. Checkpoint selection used only the validation split, and the official test remained unopened until the configuration and selected checkpoint were frozen. Class-wise instance counts are provided in Table S1.

Image identifiers preserve the relationship between related tiles. For example, lr_00001_top_left and lr_00001_bottom_right share the source-group key lr_00001, while the final terms indicate the tile position. We used this naming structure to check whether related tiles appeared in different splits. The audit found 278 shared source groups between train and validation, 344 between train and test, and 128 between validation and test. SHA-256 comparison also revealed one exact duplicate between train and test. The published splits were retained for benchmark comparability.

#### 2.3.2. Annotation and Image Preparation

Each released tile measured 2592 × 1944 pixels and had rectangular annotations in PASCAL VOC format. We converted the bounding boxes to YOLO format while retaining the six class labels. The tiles were letterboxed to 1280 × 1280 pixels during training and evaluation. We used the published tiles and splits directly. Source images were not reconstructed and no group-level split was created.

Each rectangular annotation was expressed in YOLO format as

*y* = (*c*, *x_c_*, *y_c_*, *w*, *h*), (10)

where *c* is the class index from 0 to 5. The variables *x_c_* and *y_c_* are the normalized coordinates of the bounding-box center, while *w* and *h* are the normalized width and height. All four geometric values are normalized by image width and height and lie within [0, 1].

#### 2.3.3. Training, Selection, and Evaluation Protocol

YOLOv8s was retained for its higher validation accuracy in a matched comparison with YOLOv8n. Both models used the same official training and validation splits and matched training settings, with checkpoints selected by maximum validation mAP50. At an input size of 1280 × 1280, YOLOv8n used 3.01 M parameters and 32.79 billion floating-point operations (GFLOPs) and achieved validation precision, recall, mAP50, and mAP50–95 of 0.86746, 0.79176, 0.84551, and 0.50933. YOLOv8s used 11.14 M parameters and 114.61 GFLOPs and achieved 0.88629, 0.88175, 0.90644, and 0.61563, respectively. The comparison shows a trade-off between detection accuracy and computational cost. YOLOv8n requires fewer parameters and GFLOPs, while YOLOv8s provides higher recall and mAP.

YOLOv8s was initialized from official Ultralytics COCO weights and trained for 200 epochs using the settings in Table 2. AdamW optimization used an initial learning rate of 0.001 and a cosine final factor of 0.01.

Training used seed 0, deterministic operations, and disabled automatic mixed precision on an AMD Radeon RX 9070 XT. The checkpoint with the highest validation mAP50 was selected at epoch 146 and was evaluated once on the official-test split after the configuration and checkpoint had been frozen.

Precision is the proportion of predicted detections that match ground-truth objects. Recall measures the proportion of ground-truth objects detected by the model. AP50 denotes average precision at an IoU threshold of 0.50. The mean AP50 across the six distress classes gives mAP50. For mAP50–95, AP is averaged over the six classes and ten IoU thresholds from 0.50 to 0.95, in increments of 0.05.

## 3. Results

### 3.1. LiDAR Point Cloud Deck Extraction

**Figure 3:**
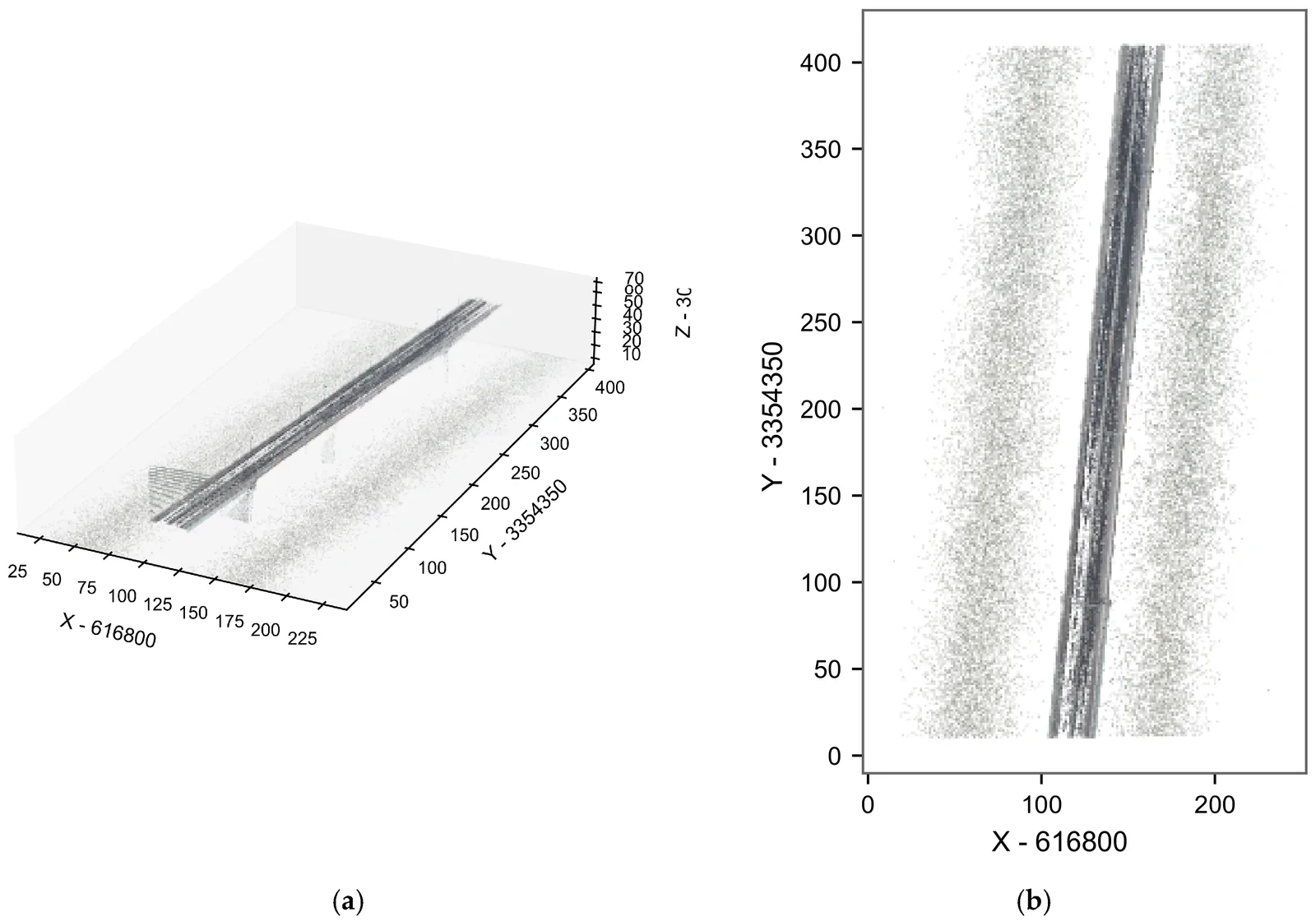
Visualization of the original bridge LiDAR point cloud: (**a**) overall three-dimensional view; (**b**) plan view. Both views show the raw point cloud before filtering, normalization, and voxel sampling. Coordinates are expressed in the original LAS coordinate units.

The LiDAR point cloud of the Jingzhou Yangtze River Highway Bridge was processed using the workflow described in Section 2.2. Figure 3 presents the raw point cloud before SOR filtering, coordinate normalization, and voxel representative sampling.

After SOR filtering, coordinate normalization, and voxel representative sampling, 311,784 representative points were retained for local geometric analysis. Effective-deck extraction subsequently retained 179,914 points according to the specified geometric criteria. The background reduction ratio was calculated as

(11) $$\mathrm{BRR}=\frac{N_{\mathrm{all}}-N_{\mathrm{deck}}}{N_{\mathrm{all}}}\times 100\%.$$

Substituting the above values gives *BRR* = 42.30%. Effective-deck extraction retained 179,914 of the 311,784 representative points. The remaining 131,870 points (42.30%) were excluded as non-deck or irrelevant background before local anomaly analysis. This metric describes point-count reduction after deck extraction. The point-count changes at each stage of the LiDAR processing pipeline are summarized in Table 3.

**Figure 4:**
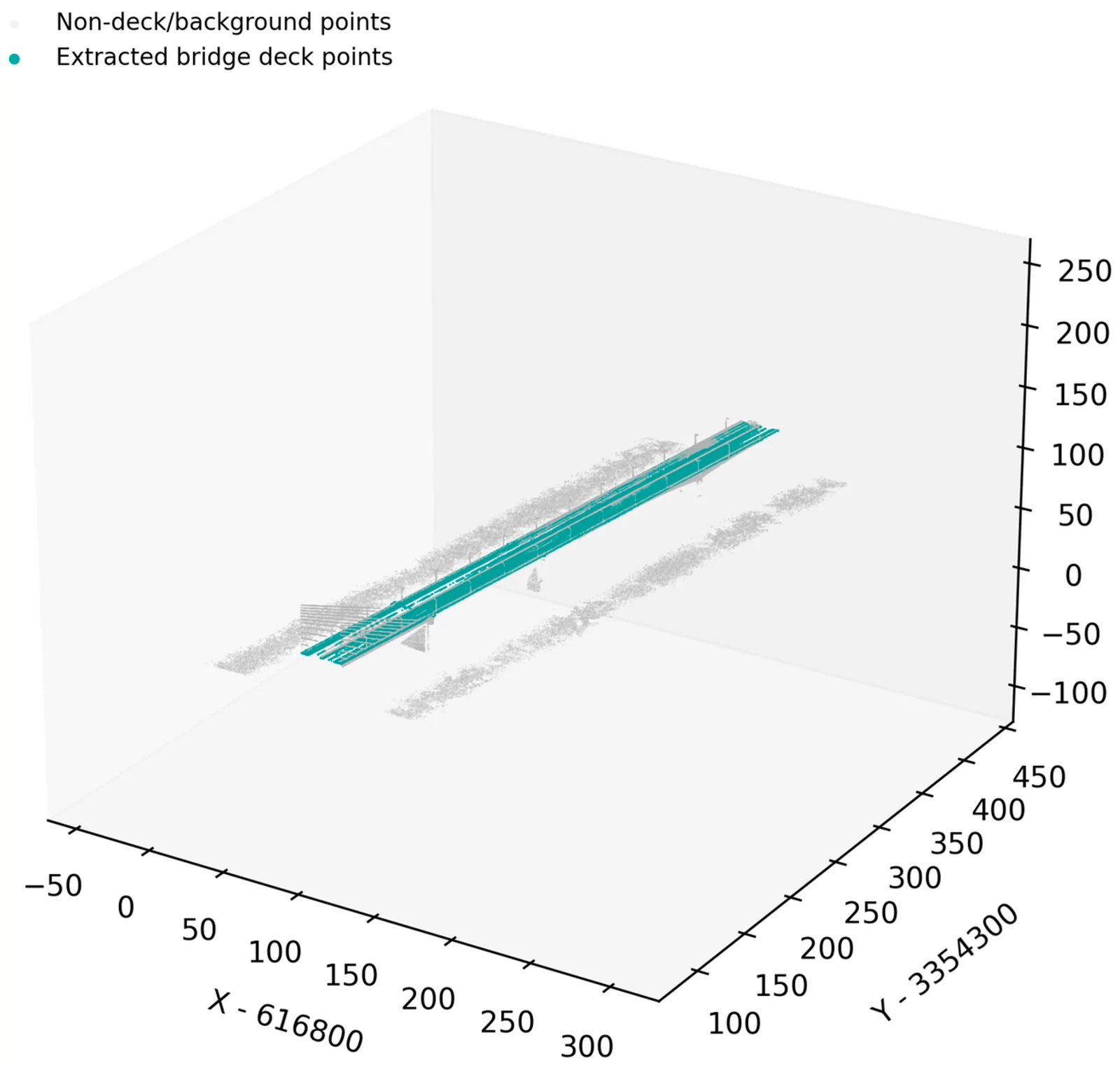
Bridge-deck extraction result from the LiDAR point cloud. Teal points indicate the extracted deck, and light-gray points provide non-deck/background context.

**Figure 5:**
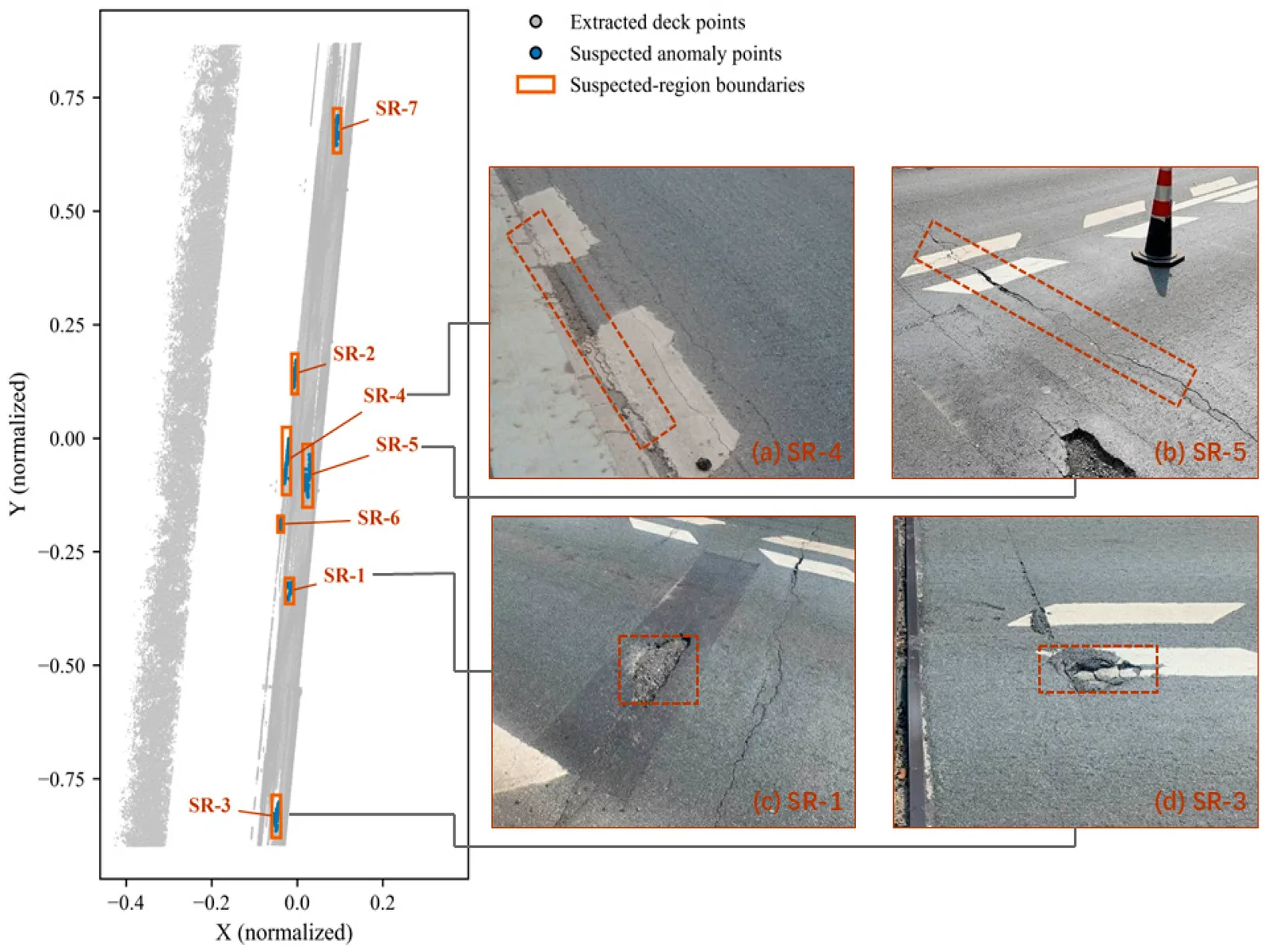
LiDAR-derived candidate regions and representative field photographs from the 2025 periodic inspection. The left panel shows the seven candidate regions (SR-1–SR-7), all of which were confirmed by site personnel to contain pavement distress. The right panels show the principal distress features in four representative regions: (**a**) SR-4; (**b**) SR-5; (**c**) SR-1; and (**d**) SR-3. Dashed boxes indicate visible pavement distress.

The bridge-deck extraction retained 179,914 points within the effective-deck area, as shown in Figure 4. Using an OR rule, the residual and normal-discrepancy criteria identified 17,075 geometrically suspected points within the effective-deck area. These points accounted for 9.49% of the 179,914 effective-deck points. Region filtering followed by bounding-box export yielded 423 suspected points distributed across seven candidate regions. This corresponds to the removal of 16,652 initially screened points, or 97.52% of the set. The retained points formed seven spatially localized regions, as shown in Figure 5.

Within the evaluated domain D defined in Section 2.2.4, the seven candidate regions occupied 0.00329399 of 0.22521825 normalized units squared, corresponding to a candidate-area fraction of 1.463% and an area reduction of 98.537% (Table S2; Figure S1). The candidate fraction ranged from 1.400% to 1.608% across the tested grid widths and offsets (Table S3). These values describe the retained screening footprint, not distress recall or UAV flight-time savings.

Field records from the 2025 periodic inspection of the Jingzhou Yangtze River Highway Bridge were examined in relation to the seven LiDAR-derived candidate regions. Site personnel confirmed the presence of pavement distress in all seven regions and verified the regional assignments of the selected photographs. Figure 5 presents the principal distress features in four representative regions: SR-1, SR-3, SR-4, and SR-5.

These candidate regions provide spatial targets for detailed image inspection in the proposed workflow. The image-detection results reported below use the separate UAV-PDD2023 benchmark.

### 3.2. Detector Training and Overall Performance

The validation-only selected checkpoint at epoch 146 achieved a precision of 0.88629, recall of 0.88175, mAP50 of 0.90644, and mAP50–95 of 0.61563. The one-time official-test evaluation yielded 0.85591, 0.85882, 0.88556, and 0.58195, respectively. Table 4 summarizes these validation and official-test results.

**Figure 6:**
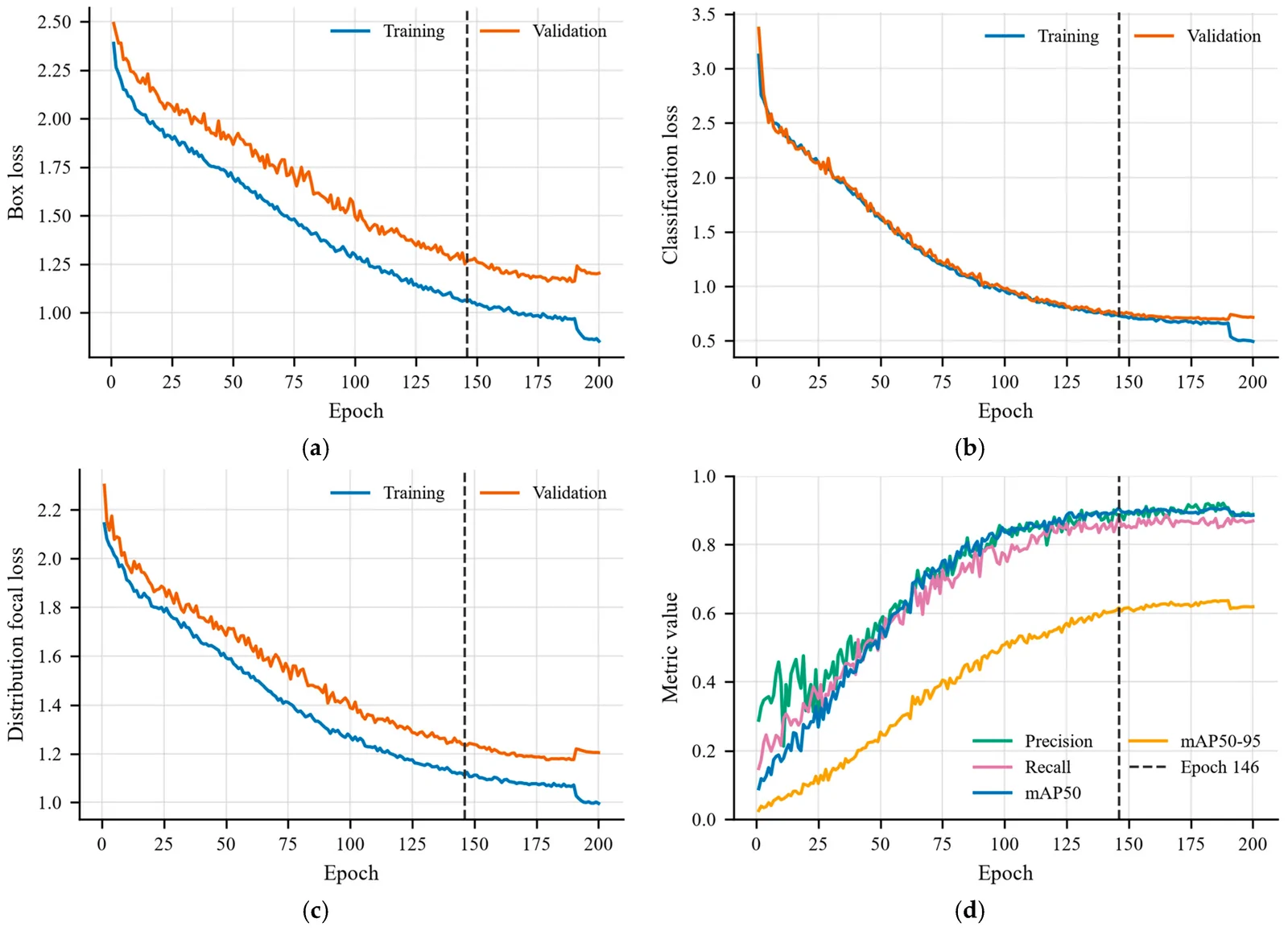
Training and validation losses and validation detection metrics across 200 epochs: (**a**) box loss; (**b**) classification loss; (**c**) distribution focal loss; and (**d**) precision, recall, mAP50, and mAP50–95. The vertical dashed line in each panel indicates epoch 146, corresponding to the selected model checkpoint.3.3. Official-Test Diagnostics and Detection Behavior.

Across 200 epochs, the training losses decreased overall (Figure 6a–c). Validation losses remained nearly stable during epochs 180–190 and increased mainly during epochs 191–200, coinciding with the scheduled disabling of mosaic augmentation for the final 10 epochs (close_mosaic = 10). The augmentation change and late-stage overfitting are possible explanations for this pattern; their individual contributions were not isolated.

Validation metrics fluctuated around a plateau during the later epochs (Figure 6d). Validation mAP50 reached its maximum of 0.90644 at epoch 146, when precision, recall, and mAP50–95 were 0.88629, 0.88175, and 0.61563, respectively. The epoch-146 checkpoint was selected for the official-test evaluation.

**Figure 7:**
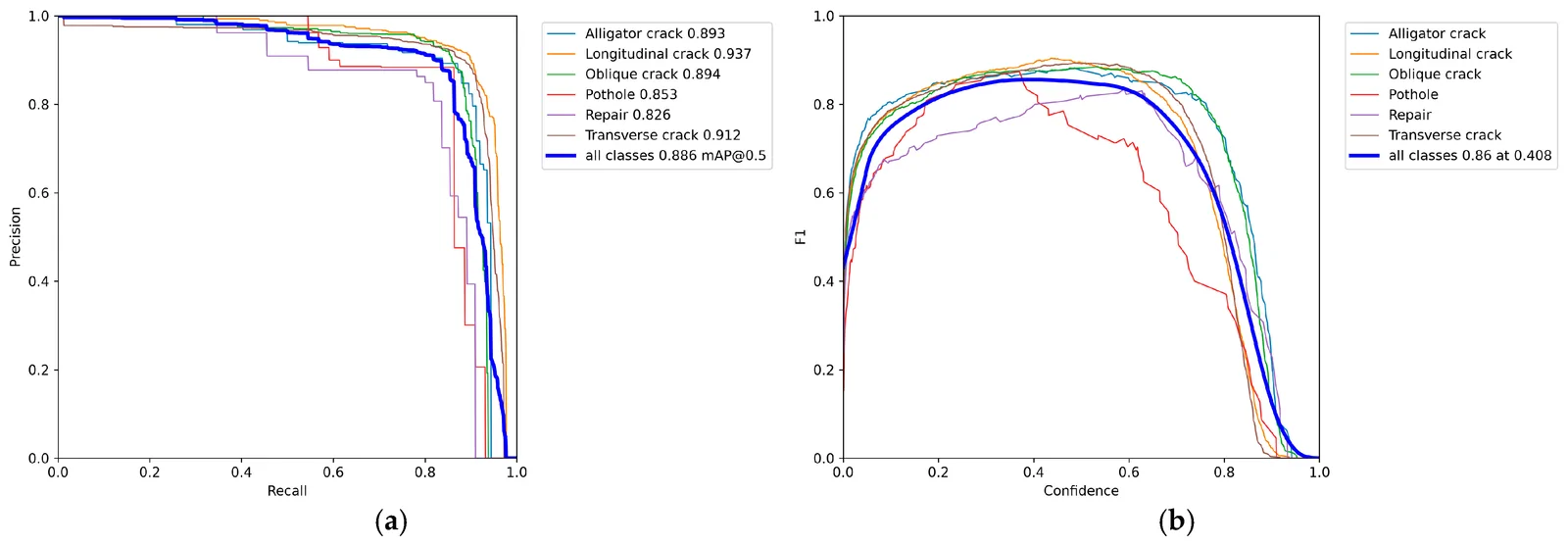
Official-test diagnostic curves for the six pavement-distress classes: (**a**) precision–recall curves; and (**b**) F1–confidence curves.

**Figure 8:**
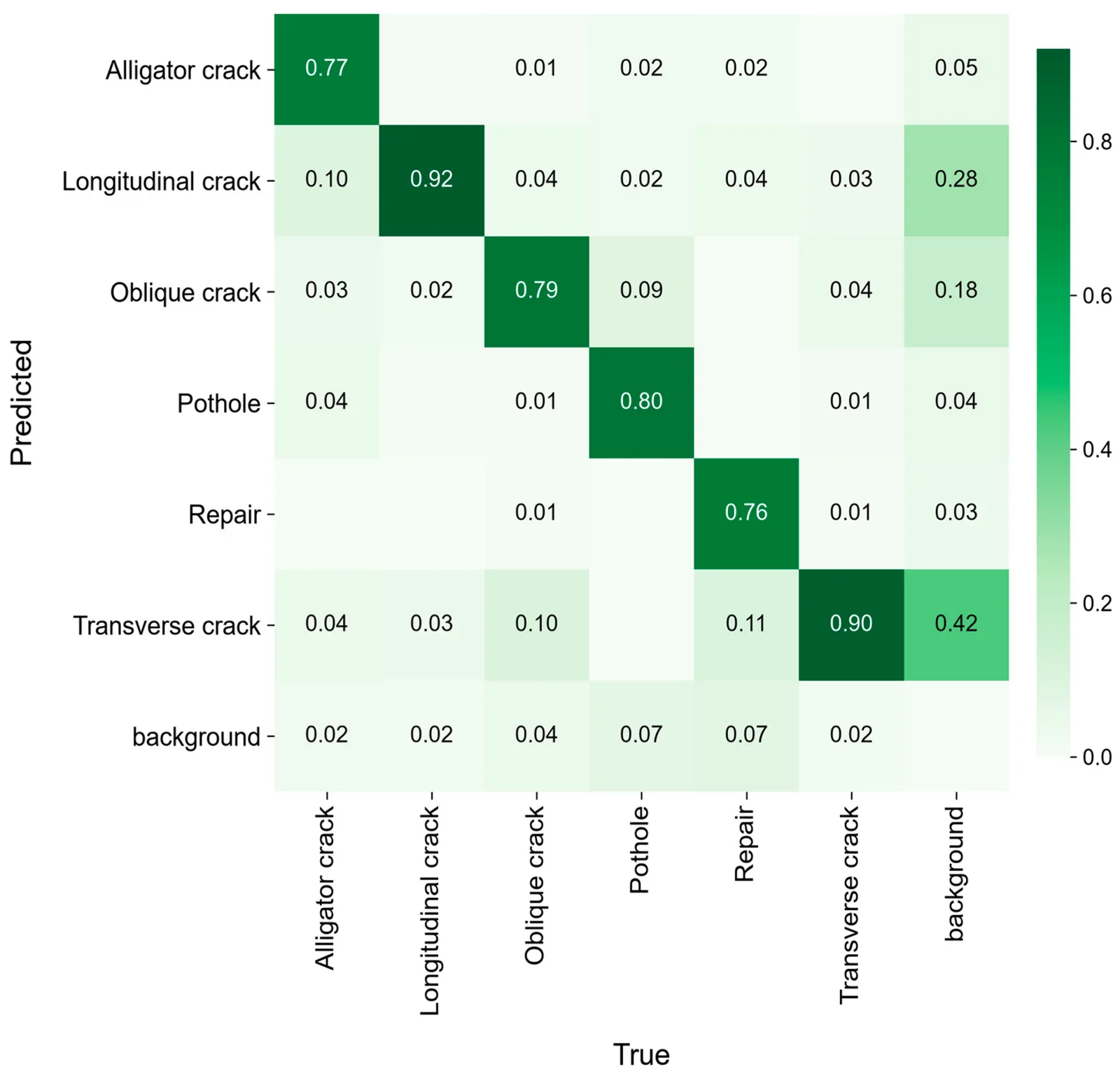
Normalized confusion matrix on the frozen official-test split.

Figure 7 shows the precision–recall and F1–confidence curves for the six distress classes on the frozen official-test split. Figure 8 presents the normalized confusion matrix for the same official-test split.

Table 5 reports per-class official-test precision, recall, AP50, and AP50–95.

**Figure 9:**
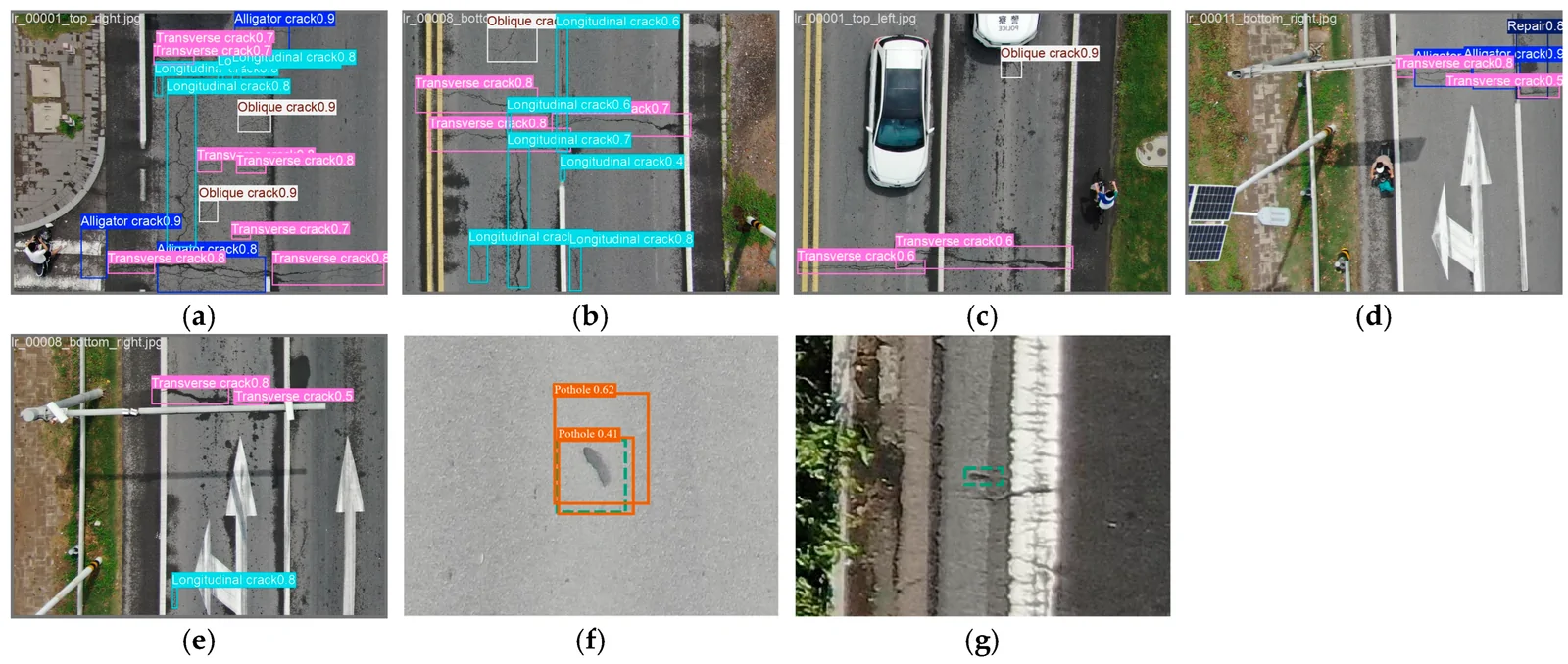
Representative official-test detections for six pavement-distress classes and one pothole miss: (**a**) alligator crack; (**b**) longitudinal crack; (**c**) oblique crack; (**d**) repair; (**e**) transverse crack; (**f**) detected pothole; and (**g**) missed pothole. Note: in panels (**f**,**g**), dashed green boxes indicate ground-truth annotations, whereas solid orange boxes indicate model predictions.

On the official-test split, pothole had the lowest recall (0.74957) and AP50–95 (0.48119), whereas repair had the lowest precision (0.76803) and AP50 (0.82566). Figure 9 presents representative detection outputs, and panel g shows one annotated pothole that the model failed to detect.

Timing was measured on an AMD Radeon RX 9070 XT with an official-test batch size of four. The Ultralytics per-image summary reported mean times of 2.45 ms for preprocessing, 26.06 ms for inference, and 0.80 ms for postprocessing. Together, these three components totaled 29.31 ms per image and covered only the model processing pipeline. Image acquisition, data transfer, and other field-operation overheads were not included. These timings describe the reported evaluation run; dedicated timing warm-up and repeated-run counts were not recorded.

## 4. Discussion

The LiDAR branch concentrates geometric screening results into spatial targets for follow-up inspection. Effective-deck extraction retained 179,914 of the 311,784 representative points and excluded 131,870 (42.30%). The two geometric criteria yielded 17,075 suspected points under the OR rule; region filtering followed by bounding-box export yielded 423 points in seven candidate regions. Earlier point-cloud assessments and curvature-based methods likewise used local geometric variation to locate anomalous deck areas [26,27]. The present workflow assigns these anomalies a screening role and reserves texture-dependent defect identification for images, consistent with the complementary sensing capabilities summarized in [28]. Existing UAV LiDAR–image fusion research [29] addresses spatially linked damage assessment. The present case uses geometric screening to define candidate regions and evaluates image detection on a separate public benchmark.

Thin cracks with weak geometric signatures may be missed during LiDAR screening. If detailed imaging covers candidate regions alone, defects outside those regions remain unexamined by the image detector.

Conversely, expansion-joint edges and drainage components can trigger geometric screening without indicating pavement distress. Raised markings or measurement artifacts may also interfere; color contrast alone is not used. The shape filters do not identify structural components. A follow-up option is to mask known component footprints using inspection records or registered imagery, while retaining adjacent pavement for review. Such masking requires separate validation.

Screening also depends on acquisition quality. The available bridge inspection report does not document the LiDAR acquisition specifications or point density over the evaluated deck domain. At lower point density, the 50-point neighborhoods span larger areas, and the 30-point cluster requirement can affect candidate retention. Ranging noise perturbs residuals and surface normals. The percentile thresholds do not establish a calibrated noise tolerance. Establishing a minimum usable density would require controlled density variation against reference annotations under a predefined performance criterion; the SOR analysis tests filtering settings rather than acquisition requirements.

Site-personnel confirmation provides qualitative support for pavement distress within all seven LiDAR-derived candidate regions. The inspection report describes defects relative to piers and deck edges, but does not link them to the LiDAR coordinate frame or establish a complete distress inventory within the evaluated domain D. These records support regional interpretation of the screening output; regional recall and a formal false-screening ratio are not quantified.

On the official-test split, potholes had the lowest recall (0.74957), as illustrated by the missed example in Figure 9g. Repair produced the lowest precision (0.76803), indicating a larger false-positive share than other classes. The smaller training sets for potholes and repairs may limit the appearance variation available for learning. Sample count alone does not explain performance: alligator cracks have 380 training instances yet achieve an AP50 of 0.89313. Across all six classes, mAP50 was 0.88556 and mAP50–95 was 0.58195. The stricter overlap requirements included in mAP50–95 make this metric more demanding of bounding-box localization. Comparisons with YOLOv8-based road and bridge studies [15,16,17,18] remain descriptive because their datasets and evaluation protocols differ.

Quantitative detector evaluation excluded the Jingzhou inspection photographs. Viewing angle, compression, sharpness, scale, and calibration were uncontrolled. UAV-PDD2023 supplied the reproducible six-class protocol used for detector training and benchmark evaluation across varied flight heights and scene compositions. Transfer to bridge decks also depends on surface context. Similar wearing-course materials can occur in both domains, but joints create different local boundaries and distress settings. Changes in repair texture or image scale may further affect recognition. Generalization to the Jingzhou bridge and readiness for routine maintenance still require calibrated images collected under consistent field conditions.

Source-group overlap and the exact train–test duplicate identified in Section 2.3.1 may inflate benchmark estimates through shared textures and backgrounds. The official splits were retained for comparability, so the reported metrics characterize this image-level protocol. Quantifying the effect of overlap requires a group-disjoint evaluation.

## 5. Conclusions

The Jingzhou case demonstrates geometric screening at bridge scale, and the UAV-PDD2023 evaluation characterizes six-class image detection under the official benchmark protocol.

(1) After geometric screening, clustering, and region filtering, the LiDAR branch retained 423 points distributed across seven candidate regions. These candidate regions provide spatial targets for follow-up pavement inspection.
(2) Site personnel confirmed pavement distress within all seven LiDAR-derived candidate regions. Figure 5 presents representative inspection photographs from four of these regions, providing regional field support for the screening output.
(3) The YOLOv8s checkpoint selected at epoch 146 achieved a precision of 0.85591, recall of 0.85882, mAP50 of 0.88556, and mAP50–95 of 0.58195 on the official UAV-PDD2023 test split.

For the next validation stage, calibrated bridge images and LiDAR points will be registered in a common coordinate system. Images withheld from training and parameter selection will be evaluated with the frozen YOLOv8s checkpoint. Precision, recall, mAP50, and mAP50–95 will measure detection performance, whereas spatial matching error will assess registration quality. Project-specific acceptance thresholds will be defined before evaluation.

## Figures and Tables

**Table 1:** Data sources, evidence scope, and analytical role.

Data Source	Evidence Scope	Analytical Role
LiDAR point cloud	10,374,647 raw LAS points from the engineering case	Deck extraction, plane-residual analysis, and candidate-region screening
2025 field-inspection evidence	The report contained defect identifiers and photographs. Site personnel confirmed corresponding distress within the candidate regions; no coordinate records were available	Regional support for interpreting the LiDAR screening results, without auditable coordinate-level matching
UAV-PDD2023	2440 annotated image tiles and 11,158 annotated instances; official train, validation, and test lists [30]	Formal detector training, validation-only checkpoint selection, and one official-test evaluation

**Table 2:** YOLOv8s training configuration and evaluation protocol.

Parameter	Value
Model	YOLOv8s
Initialization	Official Ultralytics weights pretrained on Common Objects in Context (COCO)
Official split	1561 train/391 validation/488 test images
Input size	1280 × 1280 letterbox
Epochs	200 completed
Batch size (training/validation/test)	4
Data loading	4 workers; cache = False
Optimizer	AdamW
Learning rate	lr0 = 0.001; lrf = 0.01; cosine schedule
Training warm-up	3 epochs; warmup_momentum = 0.8; warmup_bias_lr = 0.1
Randomness control	seed = 0; deterministic = True
Automatic mixed precision	AMP = False
Data augmentation	mosaic = 1.0 (disabled for final 10 epochs); mixup = 0.0; HSV h/s/v = 0.015/0.30/0.25; rotation range = −5 to +5 degrees; translate = 0.05; scale = 0.30; vertical/horizontal flip = 0.5/0.5; shear = 0; perspective = 0
Device	AMD Radeon RX 9070 XT
Software	Python 3.12.3; PyTorch 2.9.1 + rocm7.2.1; Ultralytics 8.4.113
Validation/test postprocessing	confidence threshold = 0.001; non-maximum suppression (NMS) intersection-over-union (IoU) threshold = 0.70; maximum detections = 300
Diagnostic visualization	Prediction confidence threshold ≥ 0.25; confusion matrix normalized by true class (rows: predicted; columns: true).
Timing report	Official-test batch size = 4; Ultralytics per-image summary; no dedicated timing warm-up or repeated-run count recorded
Checkpoint selection	Maximum validation mAP50 at epoch 146

**Table 3:** Quantitative results of the LiDAR branch.

Stage	Input	Output	Reduction/Share
SOR filtering	10,374,647	8,916,957	14.05% removed
Voxel representative sampling	8,916,957	311,784	3.50% retained
Effective-deck extraction	311,784	179,914	42.30% removed
Residual and normal-discrepancy screening (OR rule)	179,914	17,075	9.49% selected
Region filtering and bounding-box export	17,075	423 points in 7 suspected regions	97.52% removed

**Table 4:** Overall performance on the official validation and test splits.

Dataset Split	Precision	Recall	mAP50	mAP50–95
Validation	0.88629	0.88175	0.90644	0.61563
Official test	0.85591	0.85882	0.88556	0.58195

**Table 5:** Per-class performance on the official-test split.

Class	Precision	Recall	AP50	AP50–95
Alligator crack	0.87187	0.87804	0.89313	0.64586
Longitudinal crack	0.89117	0.90630	0.93657	0.60633
Oblique crack	0.87790	0.87252	0.89351	0.63358
Pothole	0.86835	0.74957	0.85291	0.48119
Repair	0.76803	0.83636	0.82566	0.54505
Transverse crack	0.85812	0.91014	0.91157	0.57970

## Data Availability

The UAV-PDD2023 dataset is publicly available from Zenodo Available online: https://doi.org/10.5281/zenodo.8429208 (accessed on 14 August 2026). The split-audit records, training configuration, training logs, checkpoint hash, and one-time official-test outputs generated in this study are available from the corresponding authors upon reasonable request. The Jingzhou bridge point-cloud data and periodic inspection report are not publicly available due to project ownership restrictions. Requests for access may be directed to the corresponding authors and are subject to approval by the project owner.

## Supplementary Materials

The following supporting information can be downloaded at: https://www.mdpi.com/article/10.3390/s26196217/s1, Table S1: Class-wise annotation counts in the official UAV-PDD2023 splits; Table S2: Areas of the seven saved candidate regions; Table S3: Sensitivity of the area calculation to grid width and origin offset; Figure S1: Candidate-region area accounting: (a) Occupied-cell support domain and original candidate bounds. (b) Historical display padding, excluded from area calculations. (c) Candidate intersection area as a percentage of D. The domain represents point-cloud-supported projected coverage, not an independently surveyed deck boundary. Empty cells are not connected or filled; gaps smaller than a cell cannot be resolved. The reduction describes the retained geometric footprint and does not measure distress recall or UAV flight-time savings.

## Abbreviations

The following abbreviations are used in this manuscript:

AMP	Automatic mixed precision
AP	Average precision
BRR	Background reduction ratio
COCO	Common Objects in Context
GPU	Graphics processing unit
IoU	Intersection over union
LAS	ASPRS LAS point-cloud file format
LiDAR	Light detection and ranging
mAP	Mean average precision
RANSAC	Random sample consensus
SOR	Statistical outlier removal
UAV	Unmanned aerial vehicle
YOLO	You only look once
