# Supplementary material for: A Macro-to-Micro Framework for Bridge-Deck Pavement-Distress Inspection Using LiDAR-Based Screening and YOLOv8s Image Detection: A Case Study of the Jingzhou Yangtze River Highway Bridge and Public Benchmark Data
Source: Sensors (Basel). 2026 Sep 30;26(19):6217. doi: 10.3390/s26196217 (PMC13667697; doi:10.3390/s26196217)
Supplement: Supplementary file 1 [file sensors-26-06217-s001.zip › sensors-4533744-supplementary.pdf]

Supplementary Materials

A Macro-to-Micro Framework for Bridge-Deck Pavement Distress Inspection Using LiDAR-Based Screening and YOLOv8s Image Detection: A Case Study of the Jingzhou Yangtze River Highway Bridge and Public Benchmark Data

Table S1. Class-wise annotation counts in the official UAV-PDD2023 splits.

Class	Training	Validation	Test	Total
Alligator crack	380	99	124	603
Longitudinal crack	1892	451	651	2994
Oblique crack	1070	263	353	1686
Pothole	115	36	44	195
Repair	184	43	55	282
Transverse crack	3443	831	1124	5398
Total	7084	1723	2351	11158

Counts refer to annotated instances. The training, validation, and test splits contain 1,561, 391, and 488 images, respectively. An image may contain more than one class.

Table S2. Areas of the seven saved candidate regions.

Region	Original box area	Area within D	Fraction of D (%)
SR-1	0.000435350	0.000313312	0.1391
SR-2	0.000367748	0.000166269	0.0738
SR-3	0.000812925	0.000505409	0.2244
SR-4	0.001151402	0.000458108	0.2034
SR-5	0.001430170	0.001252714	0.5562
SR-6	0.000139914	0.000088444	0.0393
SR-7	0.000667381	0.000509730	0.2263
Union	0.005004889	0.003293987	1.4626

Areas are in normalized coordinate units squared. D is the occupied-cell support domain with cell width 0.0015 and origin (0, 0); its area is 0.22521825. The candidate intersection area is 0.003293986516. Original region boxes do not overlap. Display padding is excluded.

The 17,075 suspected points formed 193 connected components. Of these, 184 contained fewer than 30 points (536 points in total). Two further components exceeded the maximum extent of 0.15 (16,131 points). Seven passed the filters before the eight-region cap, so the cap removed none. Their component membership totalled 408 points; the existing export step selected all suspected points within the saved boxes, producing 423 points.

Table S3. Sensitivity of the area calculation to grid width and origin offset.

Cell width	Offset (x, y)	Area of D	Candidate fraction (%)
0.00075	(0, 0)	0.08182800	1.5876
0.00075	(0.000375, 0)	0.08255531	1.5999
0.00075	(0, 0.000375)	0.08232019	1.5977
0.00075	(0.000375, 0.000375)	0.08318588	1.6076
0.0015	(0, 0)	0.22521825	1.4626
0.0015	(0.00075, 0)	0.21621150	1.4848
0.0015	(0, 0.00075)	0.23387400	1.4635
0.0015	(0.00075, 0.00075)	0.22305150	1.4656
0.003	(0, 0)	0.29983500	1.4142
0.003	(0.0015, 0)	0.30001500	1.4132
0.003	(0, 0.0015)	0.30035700	1.4097
0.003	(0.0015, 0.0015)	0.30021300	1.4003

The candidate fraction is area(D intersect union of candidate boxes) / area(D). Cell widths and four offsets per width were fixed before calculation. Empty cells were not filled. This is a check of boundary discretization, not a density or sensor-performance experiment.

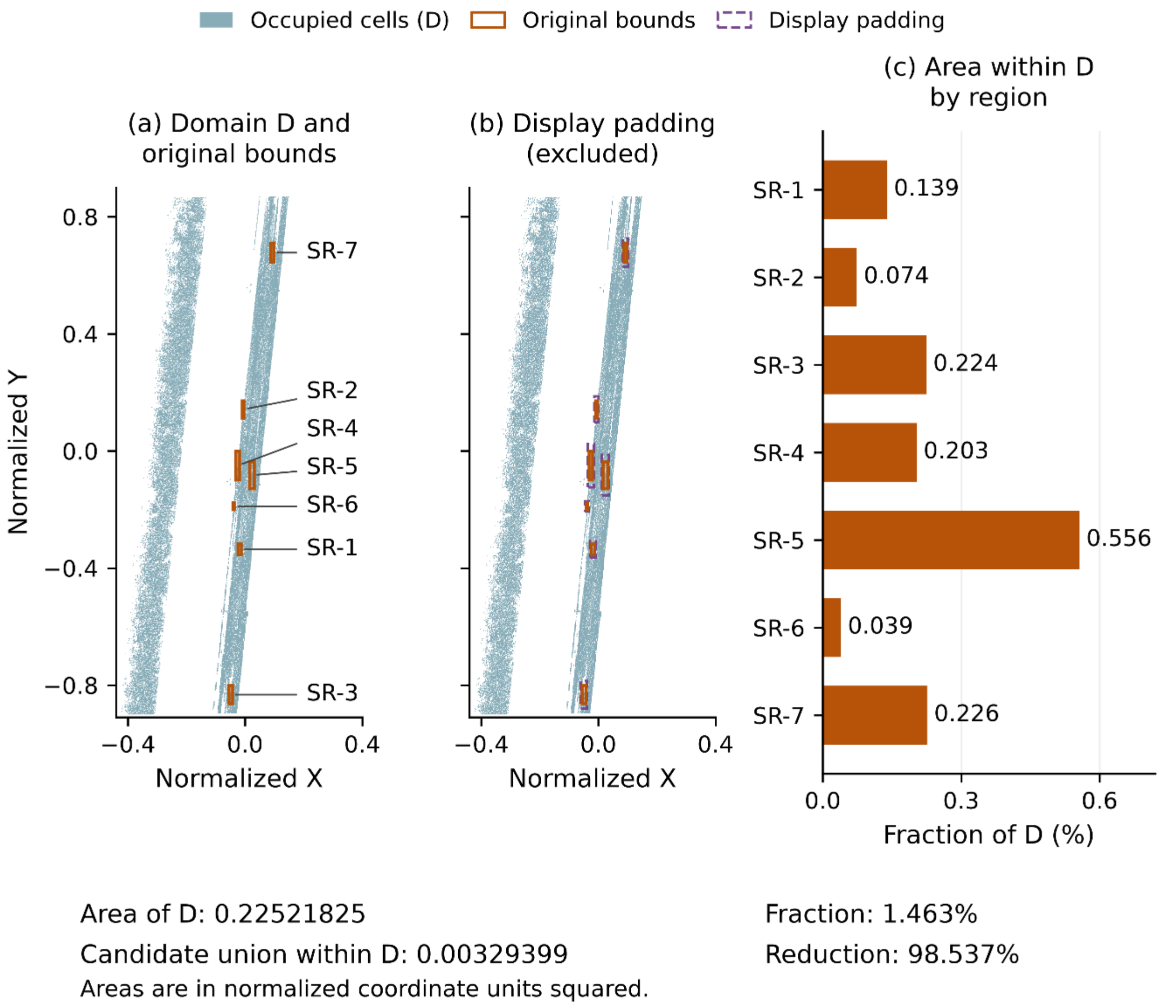

Figure S1. Candidate-region area accounting: (a) Occupied-cell support domain and original candidate bounds. (b) Historical display padding, excluded from area calculations. (c) Candidate intersection area as a percentage of D. The domain represents point-cloud-supported projected coverage, not an independently surveyed deck boundary. Empty cells are not connected or filled; gaps smaller than a cell cannot be resolved. The reduction describes the retained geometric footprint and does not measure distress recall or UAV flight-time savings.
